# Inverse Correlation Between Alzheimer’s Disease and Cancer: Short Overview

**DOI:** 10.1007/s12035-021-02544-1

**Published:** 2021-09-14

**Authors:** Agnieszka Zabłocka, Wioletta Kazana, Marta Sochocka, Bartłomiej Stańczykiewicz, Maria Janusz, Jerzy Leszek, Beata Orzechowska

**Affiliations:** 1 Laboratory of Microbiome Immunobiology, Hirszfeld Institute of Immunology and Experimental Therapy, Polish Academy of Sciences, R. Weigla 12, 53-114 Wroclaw, Poland; 2 Laboratory of Virology, Hirszfeld Institute of Immunology and Experimental Therapy, Polish Academy of Sciences, R. Weigla 12, 53-114 Wroclaw, Poland; 3 Department of Nervous System Diseases, Wroclaw Medical University, K. Bartla 5, 51-618 Wroclaw, Poland; 4 Hirszfeld Institute of Immunology and Experimental Therapy, Polish Academy of Sciences, R. Weigla 12, 53-114 Wroclaw, Poland; 5 Department of Psychiatry, Wroclaw Medical University, L. Pasteura 10, 50-367 Wroclaw, Poland

**Keywords:** Alzheimer’s disease, Cancer, Risk factors, Inflammation, Signaling pathways, Infection agents, Therapy

## Abstract

The negative association between Alzheimer’s disease (AD) and cancer suggests that susceptibility to one disease may protect against the other. When biological mechanisms of AD and cancer and relationship between them are understood, the unsolved problem of both diseases which still touches the growing human population could be overcome. Actual information about biological mechanisms and common risk factors such as chronic inflammation, age-related metabolic deregulation, and family history is presented here. Common signaling pathways, e.g., p53, Wnt, role of Pin1, and microRNA, are discussed as well. Much attention is also paid to the potential impact of chronic viral, bacterial, and fungal infections that are responsible for the inflammatory pathway in AD and also play a key role to cancer development. New data about common mechanisms in etiopathology of cancer and neurological diseases suggests new therapeutic strategies. Among them, the use of nilotinib, tyrosine kinase inhibitor, protein kinase C, and bexarotene is the most promising.

## Introduction

Alzheimer’s disease (AD) and cancer have become two of the most important global public health problems. In spite of huge progress, in both practical and theoretical medicine, the problem of their effective therapy and prevention is still unsolved.

Worldwide, nearly 50 million people have dementia, and AD affects 62% of those diagnosed with dementia. It is estimated that by 2050 the number of AD accidents will exceed 152 million in the world [1]. Another health problem, cancer, is also among the leading causes of death. It was estimated that 19.3 million new cancer cases and 10 million cancer deaths were noticed in 2020 worldwide [2].

Pathophysiological mechanisms of both cancer and AD are widely studied but yet not clearly defined. Both diseases share some risk factors. However, between the risk of developing of cancer and AD, an inverse correlation is noticed: Patients with AD show 61% decreased risk of cancer incident compared to reference subjects. A negative association can suggest the possibility that susceptibility to one disease may protect against the other.

Nevertheless, the determination how cancer can modulate the neurodegeneration and vice versa is still unsettled. A more complex understanding the underlying mechanisms linking cancer and AD will allow, not only for development of new strategies of prevention, but therapy as well.

## Biological Mechanisms in Cancer and Alzheimer’s Disease

Cancer and neurodegeneration are often thought of as disease mechanisms at opposite ends of spectrum: one due to enhanced resistance to cell death and the other due to premature cell death [3–5]. The neuropathological hallmarks of AD include senile plaques which consists of extracellular deposits of amyloid beta (Aβ) peptide and neurofibrillary tangles — intracellular deposits of an abnormally hyperphosphorylated microtubule-associated protein tau (MAPT) [6, 7]. The role in pathophysiology of AD is also played by apoptosis, synaptic loss, or neuronal dysfunction. Furthermore, oxidative stress is inextricably linked with several major pathological processes in AD [8–10]. As a final effect, neuronal loss takes place. In opposite to AD, cancer is a disorder characterized by uncontrolled, excessive cell growth [11, 12]. In many studies, the mutual protection between AD and cancer has been noticed. For example, among patients with cancer, the development of AD was reduced, and a decrease in the cancer incidence rate was observed in people with AD, in comparison to reference subjects. This inverse association could be explained by fact that multifunction mechanism that regulates cell survival is associated with these both diseases. However, the determination how cancer can modulate the neurodegeneration and vice versa is still unresolved [13].

## Association Between Cancer and Alzheimer’s Disease

Cancer and AD are both age-related multifactorial disorders. They are affected by psychosocial factors such as socio-economic status, educational attainment, and health behaviors. Molecular machinery that is involved in maintaining neural function in neurodegenerative disease may be shared with oncogenic pathways [4]. However, a comprehensive longitudinal study on large group of participants leads to the conclusion that there is an inverse association between these two diseases — the cancer diagnosis reduces the risk of subsequent AD [14–17]. The relationship between cancer and neurodegeneration is complex and several risk factors, potential mechanisms, and also both direct and inverse association, depending on the type of cancer have been reported [4]. It was shown that people with AD had a 42–50% decreased risk of incident cancer compared to reference subject. Also, the lower risk of AD (35–37%) was shown in patients with cancer [14, 18–20]. Both direct and inverse associations were noticed depending on the type of both heterogenic disorders [4]. The modestly lower AD risk in cancer patients was shown in studies of Freedman et al. [21], when cancer risk in group of AD patients were compared with automobile injuries. However, among 19 specific cancer sites examined by this group, only breast cancer, uterine cancer, and prostate cancer were statistically significantly related to a lower odds of having a previous AD diagnosis after a Bonferroni correction [21]. The relationship between cancer and risk of AD was best developed for non-melanoma skin cancer (NMSC). The long-term (more than 30 years) study on the more than 1 million of patients revealed that NMSC was associated with small reduction in relative risks of AD (5%) and all-cause dementia (8%), compared to individuals without NMSC [22]. A recent study has shown that high levels of MAPT correlate inversely with glioma aggressiveness [23]. As mentioned later, *Tau*, among other genes related to neurodegeneration, is well known for its relevance in AD [24]. It has been shown that *Tau* is also expressed in gliomas. Tau impeded the processes of angiogenesis and neo-vascularization, favoring normalization of the gliomas vasculature and therefore impeding tumor progression. As a consequence, the presence of MAPT disrupted of new blood vessels formation, which are necessary for the aggressive behavior of the tumors [23]. A nationwide cohort study using Danish population‐based health registries (1980–2013) found small inverse associations between cancer and AD which diminished over time [25]. Researchers observed a modestly reduced standardized incidence rate ratios (SIRs) after any cancer diagnosis for all-cause dementia. This inverse associations between cancer and AD were somewhat more pronounced for a cancer diagnosis in recent years. They found also diverging results by specific cancer sites, although the stage-stratified analyses were restricted by low number of dementia cases [25]. Therefore it is crucial to assess co-morbidity between these two complex diseases in association with other factors such: race, cancer type, age, and time since cancer diagnosis and environmental impact. Not only shared genetic and environmental risk factors but the role of a third disease that influences the occurrence of both AD and cancer, effects of treatment, phenotypic causality, in which one disease is a direct cause of the other disease (directional causation), or both disorders may cause one another (reciprocal causation) should be taken into consideration. Recent study on associations between cancer occurrence and AD mortality using data from population-based cancer registries from the Surveillance, Epidemiology and End Results (SEER) program has estimated the risk of AD death in cancer patients relative to reference populations stratified on demographic and clinical variables [26]. They have showed that the risk of AD death was reduced in white patients diagnosed with various cancers at 45 or more years of age, but it was increased in black patients diagnosed with cancers before 45 years of age (likely due to early onset AD). Treatment such chemotherapy decreased the risk of AD death in white women diagnosed with breast cancer at the age of 65 or more. The results were more ambiguous in case of radiotherapy which seemed to have the protective effect against AD death in women who received radiotherapy for breast cancer. However this protective effect was observed operates only early after the radiotherapy is administered [26].

It is worth mentioning that many previous studies on the relationship of AD and cancer take into account genetics factors but overlook environmental impact. This problem has been addressed in the study which examined the relationship of AD mortality to glioma mortality [27]. Researcher found that malignant brain tumors and AD in 19 US states were positively correlated. Moreover they noticed that malignant brain tumors and AD have been shown to exhibit overexpression of the same genes, for example, TREM2 (triggering receptor expressed on myeloid cells 2) [27]. One possible explanation of these results is that development of AD has been associated with environmental factors and adult malignant brain tumors simply may share part of the AD environmental risks [28, 29]. Such environmental risk factors include electromagnetic fields, hair dyes, occupational exposures to benzene, lubricating oil, wood dust, arsenic, mercury, petroleum products, lead, pesticides, and smoking [27]. Therefore the interaction of environment and genetics is complex and overlaps in case of malignant brain tumors and AD. These interesting findings need further research especially on the role of demographic (as age), biological, and lifestyle factors that could provide adequate explanations.

## Common risk Factors for Both Cancer and Neurodegeneration

**Fig. 1:**
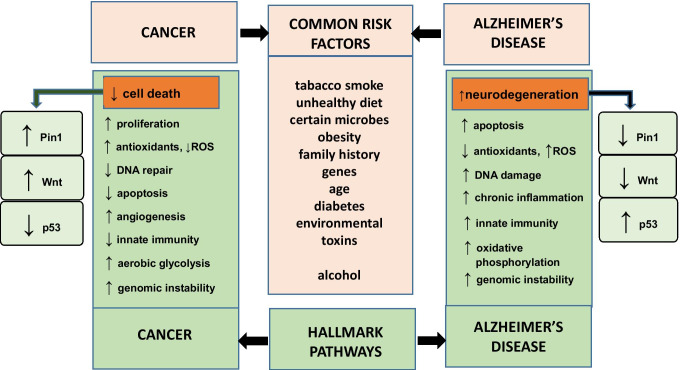
**The common risk factors, signaling pathways, and predisposition at the cellular level, for both cancer and Alzheimer’s disease.** Cancer is characterized by a decrease of apoptosis (connected to upregulated aerobic glycolysis, increased antioxidant activity, and increased proliferation). Elevation of apoptotic signals (linked with toxic protein forms, oxidative stress, inflammation or elevated oxidative phosphorylation) potentiates neurodegenerative processes and the risk of AD development. Changes which lead to suppression of Pin1 and Wnt signaling, with simultaneous p53 upregulation, increase susceptibility to neuronal death. On the other hand, upregulation of Pin1 and Wnt pathway increases the risk of tumor development

Advanced age is the most significant risk factor for both cancer and AD. A key step in their pathophysiology is inflammation and dysregulation of cell cycle. Another factors, such as diabetes, obesity, possible family history, decreased physical activity, and smoking, are also positively correlated (Fig. 1). In the both diseases, mechanisms that regulate cell survival play an important role.

Aging impacts negatively on the development of the immune system and its ability to function. Age-related metabolic deregulation and reprogramming may initiate both neurodegeneration and carcinogenesis. Both disorders are associated with pathways and genes involved in bioenergetics, inflammation, DNA damage and repair, oxidative stress, and aberrant cell-cycle activation [4, 6, 11]. In aging organisms crucial intracellular mechanisms which control cell survival, proliferation and function are dysregulated [3, 30]. Mitochondrial dysfunction is an example. The mitochondria are important organelles that regulate cell survival or apoptosis mediated by changes in the production of reactive oxygen species (ROS). ROS at physiological level can act as a second messengers to promote cell proliferation; however, higher amounts of ROS are toxic and promote apoptosis. In cancer cells, moderate production of ROS by mitochondria participates in cancer cells growth and proliferation. In AD, Aβ oligomers can be accumulated in the mitochondrial matrix and stimulate ROS production. It provides to oxidative stress, high amounts of ROS toxic to neurons are released [8, 30, 31]. Recent evidence also have shown that elevated aerobic glycolysis promotes cell proliferation and increases the risk of cancer development. In contrast, reduced glycolysis observed during aging impairs cell survival mechanisms and promotes the neurodegenerative processes [30]. Cancer and AD are both associated with aging but not often occur together in the same patients, regardless of age. The reason of unexpected clinical observation is not known, but it is that obesity-related mechanism could provide new prevention and treatment opportunities for both diseases [32].

A wide range of evidence also suggests a strong correlation between elevation of stress-related hormones such as glucocorticoids and epinephrine and development of AD hallmarks [33]. It is speculated that stress can influence on processing of amyloid beta precursor protein (APP), and subsequently on Aβ plaques formation. Additionally, it disturbs autophagy which controls the accumulation of intracellular phospho-tau and extracellular Aβ aggregation [34]. It was also shown that chronic stress can be a risk factor of cancer development [35].

To potential risk factors both for cancer and AD, chronic smoking, obesity, and diabetes are also included. The most likely mechanism underlying the association between chronic smoking and cancer and AD is vascular dementia. Obesity, together with decrease physical activity, and high-cholesterol diet are important risk factors of both cancer and AD. Negative impact of obesity on memory and cognitive function can be connected with vascular defects and impaired insulin and glucose metabolism. Epidemiological studies indicated that patients with *diabetes mellitus* are at a higher risk for developing AD and cancer. In both diseases, several metabolic abnormalities such as defected insulin signaling pathway, impairments in brain insulin responsiveness, oxidative stress, inflammation, and also abnormalities in proteins take place [36–38].

## Common Signaling Pathways in Both Cancer and AD

Aβ and intracellular deposits of hyperphosphorylated tau protein, pathological forms of proteins, disturb cellular function and provide to progressive dysfunction and loss of neurons. Cancer develops as an effect of DNA damage or intracellular stress. Moreover, the reparative processes are defective, and therefore uncontrolled cell growth takes place. In both AD and cancer, common mechanisms controlling cell survival/death are involved (Fig. 1).

The main factors controlling the surveillance mechanism are tumor suppressors. They regulate cell cycle, repair of DNA damage and protein degradation, and prevent uncontrolled proliferation under physiological conditions. Tumor suppressors also eliminate cancer cells by inducing apoptosis or cellular senescence. Among cell cycle proteins, **p53** is a particularly significant. p53 is a well-studied protein characterized by a short-life and activated in response to stress signals such as DNA damage, hypoxia, or oncogenes activation. The anticancer role of p53 is associated with stopping of cell division and compromises tissue repair [39–41]. The dysfunctional activity of p53 is common in many human cancers, e.g., leukemia, breast cancer, or gastric cancers [42, 43]. It was shown that conformational altered p53 possesses novel transcriptional features and that changes of p53 are involved in cancer development by its impact in modulation of genes responsible for encoding transcriptional modulators of oncogenic activity [44, 45].

Recent observation has been also suggested that p53 plays a crucial role both in aging and neurodegenerative disorders like AD [40, 46, 47], where an increased rate of p53 activity is correlated with aging and senescence [48, 49]. AD neuropathology which includes lethal cell cycle re-entry, excessive DNA damage, and abnormal cell death is all controlled by p53. It was shown in mice model that loss of wild-type p53 conformation reduces the regenerative capacity of the brain in response to toxic damage which confirms that p53 protein plays a crucial role in neuritogenesis and neuronal regeneration [50]. The control of synaptic genes by p53 is conserved in mammals, and that p53 is neuroprotective in an in vivo model of tauopathy [51]. Moreover, it was demonstrated that aggregates of Aβ in neurons activate p53-dependent apoptosis. Upregulation of p53 in brains of patients with AD can be also associated with the presence of changed form of presenilin 1/2 (PS1/2) [39, 46]. A strong correlation between p53 expression and excitotoxic neuronal death induced by glutamate has also been proven [38, 47]. In summary, patients with strong increase of tumor suppressors activity would be at high risk of development AD [40, 46] (Fig. 1).

**Wnt **signaling pathway plays an essential role in the control of cell survival, proliferation, and differentiation and also in maintaining carbohydrate and lipid homeostasis, gluconeogenesis, and glycolysis. It was shown to be important in the central nervous system (CNS). Wnt proteins activate at least three signaling pathways. The best understandable one is the canonical pathway that activates transcriptional activity of β-catenin/TCF transcription factor and initiate gene expression. Abnormal Wnt signaling is associated with many human diseases including cancer and neurodegeneration [47]. Aβ aggregates accumulating in the AD brain activate glycogen synthase kinase-3β (GSK-3β) and contribute in hyperphosphorylation of tau protein [52]. Same evidence also indicates that dysfunctional Wnt signaling in neurons is connected with reduction of the β-catenin level in cytoplasm and increase expression of Wnt signaling inhibitor DKK1 (Dickkopf-related protein 1). Therefore, the inhibition of Aβ_42_-mediated upregulation of GSK-3β can provide to persistent activation of Wnt signaling and can protect neurons in hippocampus against Aβ neurotoxicity. It was also shown that β-catenin level is significantly decreased in patients with AD carrying PS-1 mutations. Apolipoprotein E4 (APOE4) risk factor of AD also inhibits the Wnt signaling pathways [52]. Aβ inducing expression of DKK1 (a negative modulator of Wnt signaling) could result in increased GSK-3β activity and in consequence tau hyperphosphorylation. Therefore, the silencing or neutralizing DKK1 can activate Wnt signaling and protect neurons. It is suggested that small changes leading to suppression of Wnt signaling enhance susceptibility to neuronal death and at the same time protect against the development of cancer. On the other hand, upregulation of Wnt pathway increases the tendency to develop tumors and at the same time protects against neurodegeneration [53, 54] (Fig. 1).

The unique enzyme **Pin1 **is peptidyl-prolyl cis/trans isomerase (PPIase) that catalyzes the cis/trans isomerization of phosphorylated serine or threonine residues that precede proline. Conformational changes around the proline affect the protein structure and function. Pin 1 regulates a diverse array of central molecular processes like cell cycle, transcription, splicing regulation, DNA damage response, differentiation, or survival [49, 55, 56]. In cell cycle, regulation Pin1 exerts dual role. Firstly, it promotes G1/S transition by increasing expression and stabilization of Cyclin D1. Secondly Pin1 participates in control of DNA synthesis and centrosome duplication in phase S [57]. Additionally, Pin1 expression seems to be correlated with cell proliferative capacity: very low expression of Pin1 is observed in non-proliferating cells, while its overexpression is observed in most human cancers including the colon, breast, lung, and also brain [58, 59]. Impaired Pin1 activity has been implicated in pathogenesis of both cancer and AD. It was shown that stimulation of oncogenesis by different oncogenes is connected with prevalent overexpression of Pin1 in the most human cancers. The data published by Sherzai et al. [60] established the inverse relationship between cancer and AD based on national published data. The authors perceived that cancers with overexpression of Pin1 (such as lung or ovarian) were associates with decreased prevalence of neurodegenerative diseases such as AD.

Pin1 plays an important role in activation of multiple oncogenic pathways activated during neoplasia like Wnt or p53 [41]. Inhibition of Pin1 induces apoptosis or suppresses transformed tumor cells. It was demonstrated by Min and co-workers [61] that mice with deletion of Pin1 are resistant to cancer. The high expression of Pin1 was also shown in neuronal cells where this enzyme plays mainly neuroprotective role. Pin1 activates the Wnt/βcatenin signaling pathway, regulates of neural progenitor cells proliferation, and induces neuronal differentiation at early and late developmental stages [62]. Additionally, Pin1 control central neuronal proteins like tau or APP [55, 63]. In contract to cancer, in AD Pin1 function may be inhibited (by downregulation, phosphorylation, oxidation, or genetic changes), what leads to reduction of isomerization of tau protein and APP. The results are tau and Aβ-related pathologies and cell death. However, keeping the *trans*-conformation of tau and APP is functional and promotes normal neuron activity, *cis*-conformations, often triggers after phosphorylation, and is pathogenic. Catalyzation of isomerization from *cis* to *trans* conformation resulting in Pin1-dependent regulation of tau binding to microtubules what restore its normal function. However *cis* conformation of APP represents amyloidogenic APP processing and provides to increase production of Aβ_42._ It has been shown that cis-tau and cis-APP conformation are early pathologic species observed in mild cognitive impairment (MCI) and AD, and Pin1 activity may prevent this process [64]. In addition, it was shown that Pin-knockout mice display some aging-related AD-like features, such as neurodegeneration, tau hyper-phosphorylation, or Aβ accumulation. Interestingly, these mice were resistant to breast cancer induced by overexpression of Ras or Neu oncogene [65]. Overexpression of Pin1 restored the tau function and decreased the level of Aβ [55, 66].

## MicroRNA

MicroRNA (miRNAs) comprise one of the major, ubiquitous post-transcriptional regulatory mechanisms implicated in development, differentiation, proliferation, and apoptosis of the most eukaryotic cells. It is a class of evolutionary conserved, endogenous single-stranded non-coding RNA molecules of 21–23 nucleotides in length, which bind target mRNA to prevent translation processes. Probably, miRNAs account for 1–5% of the human genome and regulate at least 30% of protein-coding genes [67, 68]. They are one of the most promising biomarkers in the blood, due to their very high stability, and they possess pleiotropic function — the same miRNA can have beneficial or deleterious function, because it can target several mRNAs simultaneously (e.g., miRNA-34) [69]. There is a functional evidence showing that miRNAs are important for cancer-related processes and aging-related processes, due to their influence on regulating pivotal processes in the cell. For some pathways, involved in proliferation and pro-survival mechanisms, miRNAs will act opposite in AD and cancer, but for inflammation, oxidative stress and angiogenesis miRNA will possess similar functions [70]. Also, the profile of differences between AD and cancer is not strictly the same, because the miRNA profile can even depend on the tumor type and location. The inverse associations can be found, for example, between AD and hematologic malignancies, colorectal and lung cancer, but pancreatic cancer miRNA profile does not follow this rule [71].

One of the most highly expressed miRNAs in the vertebrate brain is miR-9-5p, which plays a pivotal role in the brain development. In brains of AD patients, the miR-9-5p is mostly downregulated, but there are some studies that found upregulation in the hippocampus and temporal lobe neocortex. Whereas both miR-9-5p upregulation (breast cancer, cervical cancer, glioma, gastric cancer, biliary cancer and colorectal cancer) and downregulation (melanoma and head and neck squamous cell carcinoma) have been reported in human cancers, where it can either support or suppress tumor development [69, 70, 72].

The another one, MiR-21-5p is up-regulated both in AD and cancer, but resulting in the opposite effect. It plays an important role in the oncogenic process in tumors, because it has been associated with high proliferation, invasion, and metastatic potential, as well as with low apoptosis. Oppositely, it inhibits cell apoptosis induced by Aβ, suggesting its protective role in AD [69, 73]. Downregulation of the miR-29 inhibits B cell lymphoma 2 (Bcl-2) family proteins, leads to apoptosis and it is inversely correlated with the density of amyloid plaques in brains from individuals with AD. MiR-29 was observed to be down-regulated also in cancers like melanoma, cervical cancer, endometrial serous adenocarcinoma, mantle cell lymphoma, hepatocellular carcinoma and non-small cell lung cancer. However, its downregulation results in Bcl-2 family proteins upregulation, and it promotes cell survival [70].

MiR-34a-5p has been widely recognized as a key player in tumor suppression, and its expression is silenced in several cancers. MiR-34a-5p downregulation observed in many tumors leads to enhanced autophagy, but the effect is dependent on the cancer type, stage, and location. Conversely, miR-34a-5p is overexpressed in AD patients and represses genes involved in synaptic plasticity and energy metabolism [69].

Upregulation of miR-146a-5p, one of the main miRNAs associated with cellular senescence and inflammation, leads to inflammatory response in AD but has an anti-inflammatory effect in cancer [74]. MiR-146a-5p is upregulated in AD brain and in human neural cells following a number of different stimuli and stresses, including cytokines, Aβ, and oxidative stress. In cancer, miR-146a-5p was found acting as both oncogene (cervical and anaplastic thyroid carcinoma) and oncosuppressor (prostate cancer) [69, 71].

## Inflammation in Cancer and AD

Inflammation is generated by cells in response to injury, infection, or other factors. This protective response involves immune cells, blood vessels, and molecular mediators. The function of inflammation is to eliminate the initial cause of cell injury, clear out necrotic cells and damaged tissues, and initiate inflammatory processes leading to tissue repair. In pathogen recognition and elimination processes, both innate and adaptive response play a crucial role. Innate immune cells (e.g., granulocytes, dendritic cells, macrophages, NK cells) recognize and eliminate the pathogens by toll-like receptor/nuclear factor kappa-light-chain-enhancer of activated B cells (TLR/NF-ĸB)-dependent signaling pathways. In an increased production of proinflammatory mediators, complement activation, and also enhanced recruitment and activation of lymphocytes T and B, the elements of adaptive immune take place. Innate immunity is generally non-specific, while adaptive immunity is specific to one pathogen. Although the innate and adaptive immune responses are separate defense systems, they are connected and cooperated. During acute inflammation, the innate system activates and regulates the adaptive system, but if the harmful factor act at low grade and persistent in time, this cooperation can be reversed to chronic inflammation [75, 76]. However, the inflammatory response is necessary for the elimination of pathogens, but prolonged process may lead to chronic inflammation which can provide to damage of several organs including the brain [77]. Brain inflammation is a pathological hallmark of AD. Chronic inflammation can cause DNA damage and lead to cancer. Cumulative age associated deterioration in both innate and adaptive immunity competence — immunosenescence is one of the immune mechanisms of both cancer and aging [3, 78, 79].

Inflammasomes are essential structures for host defense. They represent a critical innate immune source of interleukin 1 beta (IL-1β), a potent inflammatory cytokine, whose overproduction can contribute to autoimmune disease development. The role of inflammasomes in tumor progression is still controversial, they can show both pro- and antitumorigenic effect depending upon the type of cancer [80]. Among inflammasomes, high molecular weight protein complex, the NLR family pyrin domain containing 3 (NLRP3) is activated by aggregated Aβ. The elevated level of IL-1β, end product of inflammasome activation, has been reported in the brain of AD patients. An important role of NLRP3 inflammasome in the pathogenesis and progression of AD indicates for as attractive target for therapeutic intervention [81, 82].

The positive correlation between chronic inflammation and AD, cancer, and also diabetes was shown in many studies. The relative risks imparted by diabetes are greatest for cancers of the liver, pancreas, and endometrium. The functional relationship between inflammation and cancer is an established fact. It was observed that presence of leukocytes within tumors provided the first sign of possible link between cancer and inflammation [83]. Now, the close relationship between inflammation, innate immunity, and cancer is an accepted fact.

During the last decades, accumulating clinical and experimental data has indicated the strong arguments for promoting the role of innate immune cells in tumor progression [84]. Mechanisms by which innate immune cells potentiate tumor development are still intensively examined. It’s known that inflammatory responses play crucial role at different stages of tumor development including initiation, promotion, invasion, and metastasis. The immune system fights against tumor cells by two ways: at the intracellular level and in the cellular response mechanism that primarily affects natural killer (NK) cells. Newly transformed cells are recognized by NK cells with use of markers of specific ligands presented on tumor cells. As consequence, destruction of tumor cells takes place. Fragmentation undergoes macrophages and dendritic cells, and next tumor-derived fragments are presented to T and B lymphocytes [85]. Studies on the mechanisms of inflammatory disorders have revealed that dysregulated interactions between adaptive and innate immunity can provide to the activation of the immune system and chronic inflammation culminating in tissue damage. The cause-effect relationship is also observed in the opposite direction — the presence of cancer disease reduces immunity. It was also shown that tumor-induced changes will be manifested in reduction in total lymphocyte number, decrease in CD4 lymphocyte populations, and weakening of NK cell activity [11, 75, 83].

Inflammation is also associated with many neurodegenerative disorders including AD [86]. AD onset is associated with a complex mechanism resulting in neuronal cell death. One of the main inducer of inflammation in AD that contributes to neuronal dysfunction and death is toxic form of Aβ peptide. In the early stages of AD, neuroprotective pathways including amyloid clearance and antioxidant protection are effective [87]. Increasing with age the alterations in the production and clearance of Aβ peptide, and also its ability to aggregate into oligomers and extracellular plaques, runs the neurodegenerative processes. It was proposed that Aβ overproduction, what causes over-deposition as senile plaques, might be connected with an antimicrobial response in the brain. An antimicrobial activity of Aβ against several pathogens was noticed [88]. This observations gave a new lease of life to the infectious hypothesis of AD. Selkoe and Hardy pay attention to the important role of innate immune system in the pathogenesis of AD [89]. Intracellular molecular cascades providing to the neurodegeneration in response to Aβ aggregates and products derived from death neurons can activate microglia and astrocytes through TLR/NF-қB-dependent mechanism. It leads to the release of proinflammatory mediators like cytokines, ROS, or NO (nitric oxide). The release of high amounts of proinflammatory cytokines such as TNF-α (tumor necrosis factor alpha), IL-1β, or IL-6 (interleukin 6) may induce the apoptosis of neurons. Additionally, inflammatory mediators acting on neurons can potentiate the Aβ production and exacerbate microglia-mediated inflammatory reaction. So, communication between neurons and glia might potentiate production of neurotoxic factors and increase the development of AD and can trigger the development of chronic inflammatory response [90]. Moreover, during aging blood–brain barrier (BBB) may become more permeable for several factors including immune cells from periphery. Peripheral blood leukocytes (PBLs) which penetrate into the brain tissue may be an additional source of pro-inflammatory mediators that exacerbate neuroinflammatory state [91].

So we can see, that chronic inflammation in cancer can potentiate cancer growth and development, while in AD, it potentiates neuronal cell death and brain degeneration.

## Infectious Agents in AD and Cancer

It has been long time established that biological agents play a key role to cancer development. Even 20% of all human tumors are believed to be infection-related cancers, mainly caused by viruses but also bacteria and parasites. Relationship between variety of tumors and viruses such as human papillomaviruses (HPVs), hepatitis B virus (HBV), hepatitis C virus (HCV), Epstein-Barr virus (EBV), Kaposi sarcoma herpesvirus (KSHV, human herpes virus type 8, HHV-8), human immunodeficiency virus type 1 (HIV-1) or humanT-cell lymphotropic virus type 1 (HTLV-1), and Merkel cell polyoma virus (MCPyV) has been evidenced in many epidemiological studies [92–94]. Recent findings link also human cytomegalovirus (CMV) with breast cancer due to viral gene products were found in tumors and metastases of breast cancers [95]. Inflammatory response is involved in tumors formation due to viruses are indirect carcinogens that act via chronic inflammation. Chronic inflammatory state of infected organ more often provides a mutagenic milieu in which virus-induced genetic transformations can lead to carcinogenesis [94]. However, bacteria such as *Helicobacter pylori*, *Salmonella typhi*, *Salmonella enteritidis*, *Chlamydia trachomatis*, and their component molecules or carcinogenic metabolites, toxins, or effector proteins may also influence on their host cell inducing DNA damage and interfere intracellular signaling pathways [92].

The role of infectious agents in AD is intensively debated from the 80s last century. Since inflammation is one of the most important responses of the immune system against infections chronic viral, bacterial and fungal infections are suggested to be responsible for the inflammatory pathway in AD. It is well known that AD is associated with many non-modifiable (age, sex, genetic abnormalities) and modifiable risk factors such as obesity or diabetes, unbalanced diets, tobacco use, physical and cognitive inactivity, depression, low educational attainment, or social isolation. It is estimated that modifiable risk factors may cause up to 30% of AD cases [96]. Recently published findings indicate neuropathological changes and cognitive decline observed in AD as a manifestation of an infection in the brain or elsewhere in the body. Thus, infectious agents and their products are suspected to play a key role in AD pathogenesis as another modifiable risk factor. Although CNS is protected by the BBB system, several viral or bacterial pathogens may penetrate into the brain during life and induce inflammatory reactions. Moreover, bacterial or viral infections may increase systemic inflammatory state and the susceptibility to development of AD [97]. The best studied pathogen blamed for development of AD is herpes simplex virus (HHV-1, human herpesvirus 1). HHV-1 causes inflammation in the same areas of the brain tissue which are observed to be damaged in the early stages of AD [98]. Next to HHV-1, several other viral pathogens are suspected to be involved in AD pathology such as other members of *Herpesviridae* family and HCV. Among bacteria most important are *Spirochaetes*, *H. pylori*, or *Chlamydia pneumoniae*. Neurotropic pathogens from *Spirochaetes* infect the brain and pass into latent infection. They form biofilms which are suggested to contribute to both the chronicity and the pathogenesis of AD. Chronic spirochetal infection and their biofilms are largely responsible for the presence of Aβ, which is produced both by the microbes and by innate immune system as a response to their biofilms [99].

**Fig. 2:**
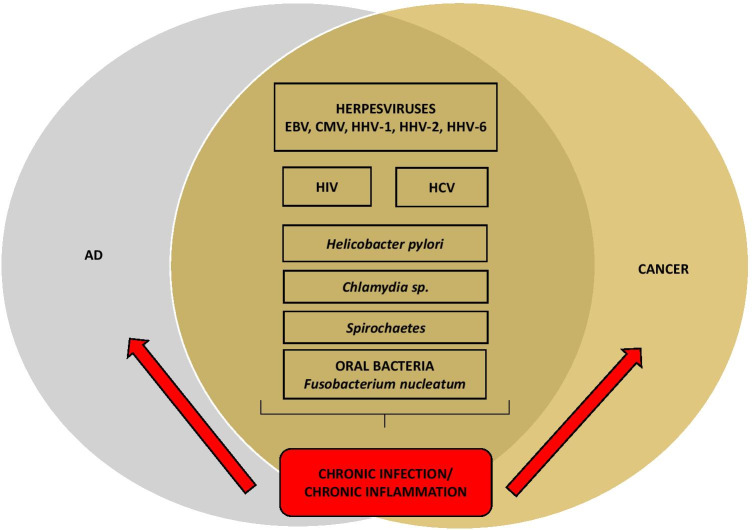
**Shared viral and bacterial pathogens for AD and cancer.** Several viral and bacterial pathogens are blamed for development and progression of cancer and AD through induction of chronic infection that in turn leads to chronic inflammatory reactions

New players, however, are oral bacteria. Oral microbiome bacteria and oral pathogens that may lead to local (gingiva, periodontium) and chronic inflammatory response are recently debated in pathogenesis of AD and cancer. In AD periopathogens such as *Porphyromonas gingivalis* are suspected to be involved in disease progression due to bacterial cells and lipopolysaccharide (LPS) were identified in AD patients’ brains [100]. The role of other periopathogens such as *Prevotella intermedia*, *Aggregatibacter actinomycetemcomitans*, *Tannerella forsythensis*, *Eikenella corrodens*, and *Fusobacterium nucleatum* has been suggested [101]. Oral cavity infections like periodontitis, if untreated, develop progressively during aging, leading to still remaining local and systemic inflammatory reactions with production of pro-inflammatory cytokines TNF-α, IL-1β, and IL-6 [102]. AD patients which suffer from periodontitis were identified to have an increased level of anti-periodontal bacteria antibodies [103]. Closely related location of the oral cavity and the brain make possibility to easy influence of oral pathogens and their products into the brain through the bloodstream or nerve fiber tracts and induce systemic immune response. Possible comorbidity between periodontitis and AD has been shown [104]. Recent studies indicate the participation of *Fusobacterium nucleatum* in the metastasis of certain types of cancer, such as colon cancer, pancreas cancer, or esophagus cancer [105]. *F. nucleatum* is a common bacterium of the oral microbiome; however, poor oral hygiene and untreated diabetes can result the bacterium cause periodontitis and tonsillitis. As a consequence, it leads to the development of inflammation. Next *F. nucleatum* may get into the bloodstream, reach cancer cells, and infect them. The association of *F. nucleatum* and cancer has been shown in studies where the DNA of the pathogen was found in colon cancer cells in greater amounts than in normal cells [106]. As a result of infection, immune responses are stimulated, which may lead to migration of tumor cells. Colon cancer cells infected with *F. nucleatum*, through the bacterial surface adhesin Fap2, have been shown to produce interleukin 8 (IL-8) and C-X-C Motif Chemokine Ligand 1 (CXCL1), which stimulate the spread of malignant cells [107]. Slade and colleagues [108] present also that these bacteria induce cytokine storm aimed to control infection, but ultimately it aggravates cancer disease. In colorectal cancer, controlling *F. nucleatum* through antibiotics and blocking *Fusobacterium*-host interactions could reduce cancer severity. It is also suggested an additional strategies based on treatment of oncogenic as well as activated host immune signaling pathways. It seems that there are possible implications of infections of the same viral and bacterial pathogens, chronic inflammation, and peripheral immune system in cancer and AD development and progression (Fig. 2).

## Promising Trends in Therapy

During treatment of cancer, cognitive dysfunction as especially short-term memory impairment, problems with attention or concentration is an increasingly recognized. Cognitive changes associated with anticancer chemotherapeutic agents are now a well-documented condition that affects some, though not all patients treated with these medications. “Chemo brain” seems to happen more often with high doses of chemotherapeutics and is more likely if the brain is also treated with radiation. Chemo brain is a phenomenon that refers to the general mental fog many patients (about 75%) with cancer experience during or after treatment [4]. The cognitive changes can negatively and sometimes dramatically impact function, quality of life, and community integration. The mechanisms underlying these cognitive changes are not fully elucidated, are still being studied, but may include direct neurotoxic effects of therapy, oxidative damage and genetic predisposition [109]. So far, there is no known way to prevent chemo brain. Interesting, there are significant differences in the occurrence of developing cognitive impairments in men and women diagnosed with cancer who received chemotherapy compared to those who did not use chemotherapy. Probably an important role lays in immune dysregulation and neurotoxicity from inflammatory cytokines. Tissue trauma and inflammation from surgery, chemotherapy, biological therapy, and targeted therapy can trigger systemic inflammation that can cross the blood–brain barrier and have deleterious effects on the CNS [110]. On the other hand, the risk of developing AD is significantly lower in patients receiving chemotherapy compared to those without chemotherapy regardless of mood disorder status [108]. Taxanes stabilize microtubules and have been proposed as potential therapeutic agents for AD [111]. In animal studies, the APOE-directed cancer chemotherapy drug bexarotene is effective in clearing Aβ from the brain of mouse model of AD and also improving their condition [112]. The cancer drug carmustine with chronic administration also reduced Aβ generation and plaque burden in mice [113]. These observations have led to interest in repurposing oncology drugs for the treatment of AD [114]. For example, if proteasome inhibitors are effective against cancer, then proteasome activators, including antioxidants, may be effective against neurodegeneration [14]. According to clinical observations, there are a significant association between chemotherapy and risk of developing drug-induced dementia of AD in patients with some kind of cancer, e.g., colorectal cancer, but chemotherapy is associated with a decreased risk of other dementias [109].

It is interesting that AD might be similar to brain cancer. The nerve cells in affected regions of the Alzheimer’s brain looked like they were trying to divide several of the proteins characteristic for cancer cells seem to be expressed at higher level in the nerve cells [115]. Researchers now are looking for development of anti-cancer treatment that can be taken as a prevention before symptoms of AD develop. So far, the results were seen only in animal models, but it is hoped that drug which could intensify the natural immune response in humans and prevent the build-up of toxic amyloid clumps could be the key tendency of AD treatment [116]. One of the more interesting and helpful drugs is bexarotene used in patients with non-Hodgkin lymphoma. A team from the University of Cambridge showed on the molecular level that this drug stops the first stages of primary nucleation. This process take place when naturally occurring proteins “misfold” themselves and clump together with other proteins to form thin filament-like structures called amyloid fibrils, and smaller protein clusters called oligomers [117]. The answer for question what is the exact mechanism, how and why the nerve cells in AD brain decide to turn on their cell division system, and what is exactly mechanisms of bexarotene action in AD is still waiting for elucidation. A recent clinical trial of bexarotene in people with Alzheimer’s was not successful, but this new work in worms suggests the drug may be given at very early step of the disease development [18, 117, 118]. Among the most promising drugs tested, both in cancer and AD are tyrosine kinase inhibitors. These drugs block the action of factors involved in cancer cell signaling, growth, and division [119]. Nilotinib (Tasigna TM) a Food and Drug Administration (FDA)-approved tyrosine kinase inhibitor used to treat leukemia is thought to promote the removal of Alzheimer’s associated proteins, such as beta-amyloid and tau. In preclinical studies have been found that nilotinib reverses cognitive impairment and reduces toxic protein deposits in animal models of Alzheimer’s [120]. Another protein which shows an important link to AD is protein kinase C (PKC). This protein acts as an information processor, or signal transducer. Excessively active PKC is associated with AD and with cancer progression, too, it can be used as potential therapeutic target in both diseases. Lastly, it has been found that the 37/67 kDa high-affinity laminin receptor (laminin receptor precursor/laminin receptor, LRP/LR) plays an important role not only in the malignancy of various cancer types but also in facilitating the processes leading to neurotoxicity in AD. Molecular techniques (such as specific antibodies and RNAi methodologies) directed against LRP/LR could prove to be effective in the prevention of metastasis but also play an important role in the production and internalization of the neurotoxic Aβ peptides in AD [121]. In vivo animal trials testing the possibility of using this antibody as a therapeutic for the treatment of AD have been initiated in transgenic mouse model.

It has to be mentioned that some recently published studies noted potential therapeutic effect on tumors of different types and AD as well. For example, Song et al. [122] reviewed that myricetin which possesses some biological activities such as antitumor, anti-inflammatory inhibits the proliferation of various cancer cells (e.g., the liver, breast, ovarian, colon, thyroid, prostate, lung cancer, leukemia, glioma, human placental choriocarcinoma) and has anti-neurodegenerative activity. Tavares et al. [123] pointed that mercaptoacetamide-based histone deacetylase inhibitors have some anti-tumor effects (e.g., prostate cancer) and might be considered as a potential therapeutic target for neurodegenerative disorder’s such as AD due to promoting dendritic spine density, leading to decrease in human Aß40, Aß42, and phosphorylated tau (Thr181) levels, and impacts Aß levels by downregulating Aß-production pathways while upregulating Aß clearance pathways. Cerium oxide nanoparticles (CNPs) have also anti-cancer properties and protects against Alzheimer’s disease. In vivo research showed that CNPs might reduce tumor growth and angiogenesis in melanoma, ovarian, breast, and retinoblastoma cancer cell-induced mice. Moreover, CNPs linked with triphenylphosphonium or magnetite nanoparticles reduced Aβ, glial fibrillary acidic protein, inflammatory, and oxidative stress markers in mice [124]. Importantly, it has been determined that Alzheimer’s drug memantine triggered bcl 2-like protein (Bax) 4-dependent pathway of apoptosis in 4T1 breast cancer cells and inhibited p-ERK protein expression in a time dependent manner [125]. Additionally, the considerable efforts have been made to conceptualize the dietary phytochemicals and their relation to oxidative stress and human diseases [126]. This recently published résumé revealed that a functional diet rich in polyphenols, fatty acids, alkaloids, and lycopene is helpful to alleviate neurological and cognitive diseases and a broad number of phytochemicals delay the spread and growth of cancer cells as well [126]. Similarly, Mandlik and Namdeo [127] showed that numerous studies have reported the neuroprotective effects and pivotal role in the prevention and treatment of several kinds of cancers (e.g., breast, renal, lung cancers) of *Withania somnifera*. Diallyl disulfide (DADS) is one of the major volatile degradative garlic compounds and some neuroprotective effects in animal models of AD and anti-cancer (e.g., inhibition of oxidative stress, cellular apoptosis, angiogenesis, and GSK-3β/NF-κB-associated signaling) has been also established [128]. These results conducive to conclude that the development of new drugs which target for both disorders is possible.

In spite of huge progress in both theoretical and practical medicine, the prevention and effective therapy of cancer and AD the main destructive disorders which affect still growing human population, are still waiting for definitive solution.

## Conclusion

There is convincing epidemiologic and scientific evidence linking the inverse cancer comorbidity with people with Alzheimer’s disease, and indicating the contribution of many etiological factors and pathophysiological processes. The relationship between cancer and neurodegeneration is complex and several risk factors, also both direct and inverse association, depending on the type of cancer have been reported. However, the additional studies should be carried out to establish the this relationship, with penetrating analyses applied to determine whether this phenomenon links to different cancer types and subsequent cancer treatment. Further experimental studies are necessary to explain how AD conditions may influence tumorigenesis and also to determine how anti-cancer drugs could modulate AD-type pathology. Advanced age is the most significant risk factor for both cancer and AD, and it negatively influences the development of the immune system and its ability to function. In the pathogenesis of these diseases, chronic dysregulation of cell cycle, inflammation, and immunosenescence is also involved. Moreover, other factors, such as diabetes, obesity, possible family history, decreased physical activity, and smoking, are also positively correlated. Different biological processes such as impaired cell proliferation and survival pathways have been suggested to have an important role underlying this inverse association. Common biological mechanisms, e.g., Pin1, Wnt, or p53 signaling, operating in inverse mode in this two disorders, lead to uncontrolled cell growth and survival in cancer or to the apoptosis and neurodegeneration in AD. In turn analysis of the research on the role of microRNA in AD and cancer indicated its ability to regulate cancer-related and aging-related processes and included a variety of important molecular mechanisms in cancer that could potentially contribute to AD pathology. Nowadays, much attention is also paid to the potential impact of chronic viral, bacterial, and fungal infections that are responsible for the inflammatory pathway in AD and also play a key role in cancer development. Recently published data suggest that susceptibility to cancer may protect against neurodegeneration, and vice versa. Therefore, a better understanding of the of the basis for this inverse relation may lead to the development of novel therapies and should remain a focus of intense basic and translational research. New data about biological mechanisms in etiopathology of cancer and AD can reveal new directions in the treatment and preventing of both diseases. Among them the use of taxanes, bexarotene, cerium oxide nanoparticles, or nilotinib, tyrosine kinase inhibitor, are the most promising.

## Data Availability

Not applicable.

## Abbreviations

AD: Alzheimer’s disease
APOE4: Apolipoprotein E4
APP: Amyloid precursor protein
Aβ: Amyloid beta
Bax: Bcl2-like protein
BBB: Blood–brain barrier
Bcl2: B cell lymphoma 2 family protein
CMV: Human cytomegalovirus
CNPs: Cerium oxide nanoparticles
CNS: Central nervous system
CNS: Central nervous system
CXCL 1: C-X-C motif chemokine ligand 1
DADS: Diallyl disulfide
DKK1: Dickkopf-related protein 1
EBV: Epstein-Barr virus
GSK-3β: Glycogen synthase kinase—3β
HBV: Hepatitis B virus
HCV: Hepatitis C virus
HHV-1: Human herpes virus 1
HIV-1: Human immunodeficiency virus type 1
HPVs: Human papillomaviruses
HTLV-1: Human T-cell lymphotropic virus type 1
IL: Interleukin
KSHV: Kaposi sarcoma herpesvirus
LPS: Lipopolysaccharide
LRP/LR: Laminin receptor precursor/laminin receptor
MAPT: Microtubule associated protein tau
MCI: Mild cognitive impairment
MCPyV: Merkel cell polyoma virus
MiRNAs: MicroRNA
NF-kB: Nuclear factor kappa light-chain-enhancer of activated B cells
NK cells: Natural killer cells
NLRP3: NLR family pyrin domain containing 3
NMSC: Non-melanoma skin cancer
NO: Nitric oxide
PBLs: Peripheral blood leukocytes
PD: Parkinson’s disease
PDD: Parkinson’s diseases dementia
PKC: Protein kinase C
PPIase: Peptidyl-prolyl cos/trans isomerase
PS 1/2: Presenilin 1/2
ROS: Reactive oxygen species
TLR: Toll-like receptor
TNF-α: Tumor necrosis factor alpha
TREM2: Triggering Receptor Expressed on Myeloid Cells 2
